# Characteristics of patients with cryptogenic stroke and atrial fibrillation detected using insertable cardiac monitoring in the chronic phase: a subanalysis of the LOOK study

**DOI:** 10.3389/fneur.2026.1795198

**Published:** 2026-05-15

**Authors:** Takehiro Katano, Satoshi Suda, Masafumi Morimoto, Yoshifumi Tsuboi, Ryosuke Doijiri, Kazutaka Sonoda, Masatoshi Koga, Masafumi Ihara, Yasuyuki Iguchi, Hidetomo Murakami, Yukako Yazawa, Kazumi Kimura

**Affiliations:** 1 Department of Neurology, Nippon Medical School, Tokyo, Japan; 2 Department of Neurosurgery, Yokohamashintoshi Neurosurgical Hospital, Yokohama, Kanagawa, Japan; 3 Department of Neurosurgery, Kawasakisaiwai Hospital, Kawasaki, Kanagawa, Japan; 4 Department of Neurology, Iwate Prefectural Central Hospital, Iwate, Japan; 5 Department of Neurology, Saiseikai Fukuoka General Hospital, Fukuoka, Japan; 6 Department of Cerebrovascular Medicine, National Cerebral and Cardiovascular Center, Osaka, Japan; 7 Department of Neurology, National Cerebral and Cardiovascular Center, Osaka, Japan; 8 Department of Neurology, The Jikei University School of Medicine, Tokyo, Japan; 9 Division of Neurology, Department of Internal Medicine, Showa Medical University School of Medicine, Tokyo, Japan; 10 Department of Stroke Neurology, Kohnan Hospital, Sendai, Miyagi, Japan; 11 Division of Research of Stroke Treatment, Department of Neurology, Kumamoto University Hospital, Kumamoto, Japan

**Keywords:** atrial fibrillation, cryptogenic stroke, embolic stroke, insertable cardiac monitor, large-vessel occlusion

## Abstract

**Introduction:**

Occult atrial fibrillation (AF) is a major underlying cause of cryptogenic stroke (CS) and is frequently identified only after hospital discharge. However, the characteristics of late-detected AF remain unclear. In this study, we aimed to investigate the clinical characteristics of patients with late-detected AF identified >30 days after the onset of stroke, compared with those without AF detection.

**Methods:**

Data from patients with CS who underwent implantation of an insertable cardiac monitor in the multicenter LOOK study were analyzed. Patients were categorized into three groups. Early-detected (within 30 days), Late-detected (after 30 days), and No-detected AF. Clinical characteristics were primarily compared between the Late-detected and No-detected AF groups, with additional exploratory comparisons involving the Early-detected AF group.

**Results:**

AF was detected in 42 (32.8%) of the 128 patients (median age, 71 years; 68.0% male). Eight patients were classified into the Early-detected AF group, 34 into the Late-detected AF group, and 86 into the No-detected AF group. Compared with the no-detected AF group, patients in the late-detected AF group were older (78 vs. 66 years, *p* < 0.001) and had lower hyperlipidemia rates (17.6% vs. 39.5%, *p* = 0.03), but higher rates of large-vessel occlusion (26.5% vs. 10.5%, *p* = 0.01) and D-dimer levels (1.1 vs. 0.7 μg/mL, *p* = 0.04). The modified Rankin Scale score before insertable cardiac monitor implantation tended to be lower in the late-detected AF group than in the early-detected AF group (*p* = 0.05).

**Discussion:**

Patients with CS and late-detected AF, compared with those without AF detection, were older, had fewer atherosclerotic risk factors, and more frequently had large-vessel occlusion. These findings highlight the importance of long-term rhythm monitoring after stroke in patients at higher risk of late-detected AF.

## 1 Introduction

Cryptogenic stroke (CS) accounts for approximately one-quarter of all ischemic strokes (1), and detecting occult atrial fibrillation (AF) is essential for effective secondary prevention (2–4). Paroxysmal AF is not always identifiable at the onset of stroke, and conventional short-term electrocardiographic monitoring does not usually detect AF. Consequently, long-term rhythm monitoring using an implantable cardiac monitor (ICM) has become increasingly utilized, and several studies have revealed that ICMs are superior to standard monitoring for identifying occult AF (5–8).

Most previous studies using ICM have focused primarily on AF detection; however, the timing of AF detection has received relatively little attention (5–8). In real-world clinical practice, continuous cardiac monitoring is typically performed during hospitalization, whereas surveillance is less intensive post-discharge. Therefore, undetected AF in the acute phase is often identified during long-term follow-up. Indeed, several studies have reported that occult AF is more frequently detected during extended monitoring than during the early post-stroke stage (5, 9–11). Recently, a systematic review and meta-analysis revealed that earlier implantation of an ICM after a cryptogenic or embolic stroke of an undetermined source was associated with a higher overall AF detection and a shorter time to diagnosis, highlighting the clinical importance of monitoring the timing of post-stroke AF detection (12).

Early-detected AF, captured shortly after the onset of an ischemic stroke, is more likely to be identified through routine short-term monitoring (5, 6, 9, 10). Conversely, AF detected in the post-stroke late phase is often more challenging to capture, owing to limited understanding of the characteristics of patients in whom it is detected after the early period. Therefore, identifying patients at high risk for late-detected AF may enable more efficient use of insertable cardiac monitors and improve post-discharge monitoring strategies. In this study, we aimed to investigate the clinical characteristics of patients with late-detected AF identified >30 days after the onset of stroke, compared with those without AF detection.

## 2 Materials and methods

### 2.1 Study design and population

This was a predefined subanalysis of the multicenter, prospective LOOK study, which investigated AF detection in patients with CS using ICM. The overall study design and protocol have been described previously (13).

From the patients enrolled in the LOOK registry, we enrolled those who underwent ICM implantation within 30 days of the index stroke. Patients who withdrew consent or were lost to follow-up were excluded. Moreover, to ensure a strict comparison of AF detection timing from the onset of stroke, patients who received ICM after 30 days were excluded from this sub-analysis based on a predefined criterion.

This study was reviewed and approved by the Ethics Committee of Nippon Medical School and the institutional review boards of the participating hospitals in the LOOK study. Written informed consent was obtained from all the enrolled patients.

### 2.2 Definition of cryptogenic stroke

CS was diagnosed according to the Japanese guidelines for CS and Embolic Stroke of Undetermined Source, requiring the exclusion of major cardioembolic sources, branch atheromatous disease, and other determined etiologies through a standardized diagnostic workup. All patients underwent magnetic resonance imaging, electrocardiographic monitoring for ≥24 h, transthoracic and transesophageal echocardiography, vascular imaging, and laboratory testing before ICM implantation unless contraindicated.

### 2.3 ICM monitoring and AF definition

All patients received Reveal LINQ ICM (Medtronic, Minneapolis, MN, USA). Data were monitored through the CareLink Network and independently reviewed by at least two physicians, with final adjudication by a board-certified cardiologist when necessary.

AF was defined as an irregular rhythm without P waves lasting ≥30 s. The timing of AF detection, ICM implantation, and recurrent stroke was measured from the onset of stroke.

### 2.4 Classification into early-detected, late-detected, and no-detected AF groups

Patients were categorized into three groups according to the time from the onset of stroke to the first AF detection: Early-detected AF group (AF detected within 30 days); Late-detected AF group (AF detected after 30 days and within 24 months), and No-detected AF group (No AF detected during the 24-month follow-up). The 30-day cutoff was based on prior trials (5, 6) and guideline recommendations for short-term monitoring of CS.

### 2.5 Study objectives

The primary objective of this study was to identify clinical characteristics associated with late-detected AF by comparing patients with late-detected AF and those without AF detection.

A secondary exploratory analysis was performed to compare clinical characteristics between patients with late-detected AF and those with early-detected AF to assess potential differences in timing-related phenotypes.

### 2.6 Clinical variables

The collected data included demographics, vascular risk factors, National Institutes of Health Stroke Scale score, modified Rankin Scale score, radiological, electrocardiographical, transthoracic, and transesophageal echocardiographic findings, and laboratory biomarkers. Large-vessel occlusion (LVO) was defined as occlusion of the internal carotid artery, the M1 segment of the middle cerebral artery, the basilar artery, or the vertebral artery on baseline vascular imaging. The data collection protocol followed the original LOOK study design.

### 2.7 Statistical analysis

Continuous variables were presented as mean ± standard deviation or median with interquartile ranges and compared using the Mann–Whitney U test or an appropriate parametric test. Categorical variables were compared using the chi-squared test or Fisher’s exact test.

For the primary analysis, clinical characteristics were compared between the Late-detected AF and No-detected AF groups. Variables with *p* < 0.05 in univariable analyses were entered into a multivariable logistic regression model to identify independent predictors of late-detected AF. Odds ratios (ORs) and 95% confidence intervals (CIs) were calculated.

For the secondary exploratory analysis, clinical characteristics were compared between the Late-detected AF and Early-detected AF groups using univariate statistical methods. Given the small sample size of the Early-detected AF group, these analyses were considered exploratory and hypothesis-generating, and no multivariable analysis was performed.

Kaplan–Meier analysis was used to estimate cumulative AF detection. Statistical significance was defined as two-sided *p* < 0.05. Variables with *p* < 0.05 in the univariate analysis were considered candidates for multivariable analysis.

To avoid overfitting, only the clinically relevant variables were included in the final model.

All analyses were performed using SPSS version 29 (IBM Corp., Armonk, NY, USA).

## 3 Results

**Figure 1:**
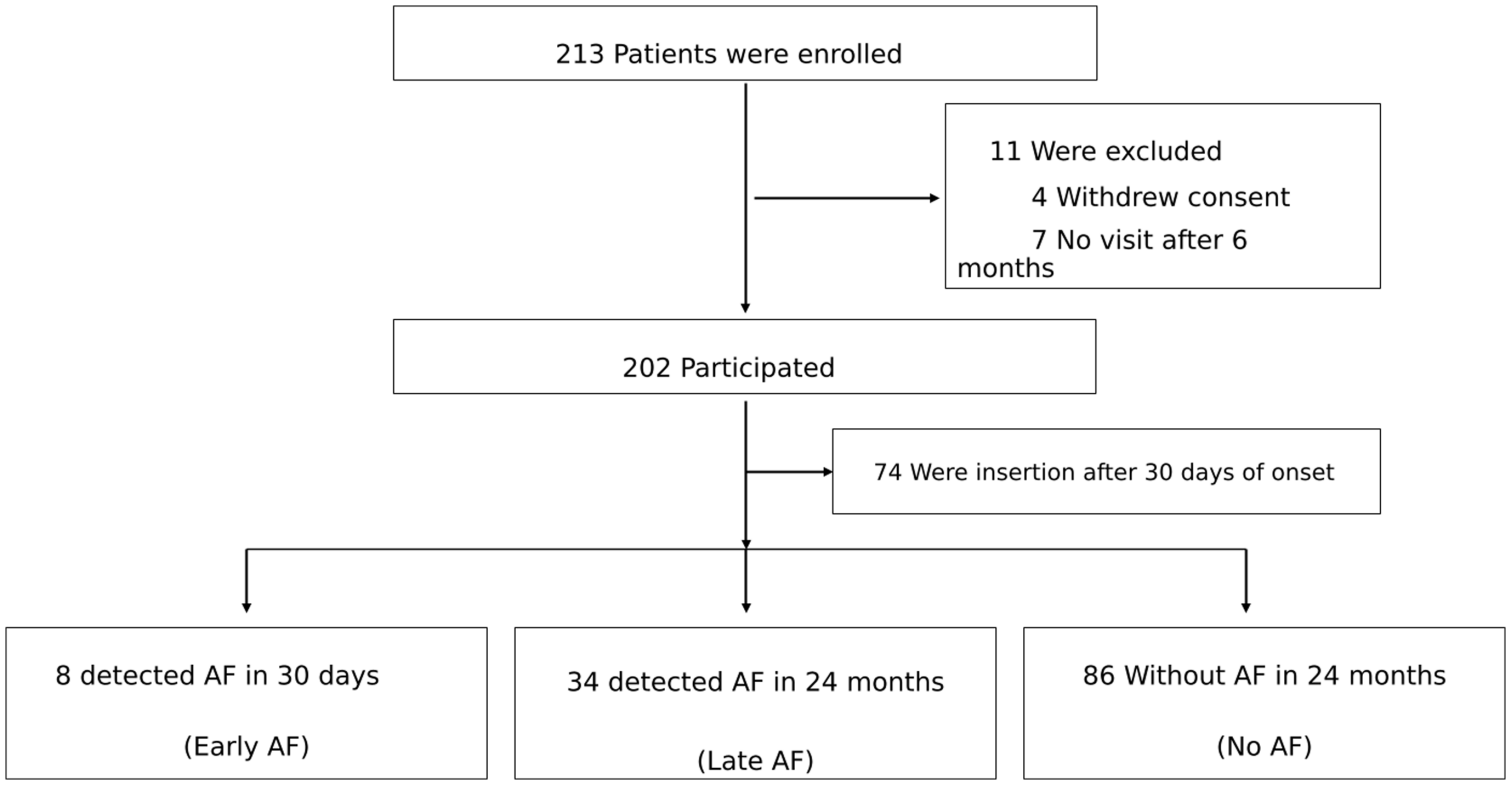
Study flow diagram of the LOOK sub-analysis. A total of 213 patients were initially enrolled in the study. Eleven patients were excluded, including four who withdrew consent and seven who were lost to follow-up, leaving 202 eligible patients. Among them, 74 patients underwent insertable cardiac monitor (ICM) implantation more than 30 days after stroke onset and were excluded. Consequently, 128 patients were included in the present analysis. During the 24-month follow-up period, atrial fibrillation (AF) was not detected in 86 patients (No-detected AF group), while AF was detected in 42 patients: 34 patients were classified as the Late-detected AF group (AF detected after 30 days) and 8 patients as the Early-detected AF group (AF detected within 30 days after stroke onset).

Overall, 4 of the 213 consecutive patients enrolled in the LOOK study withdrew consent, and 7 were lost to follow-up, leaving 202 eligible cases. Among them, 74 underwent ICM implantation after 30 days. Therefore, 128 patients were included in this study. Patients were categorized into three groups: 8 in the Early-detected AF group (AF detected within 30 days), 34 in the Late-detected AF (AF detected after 30 days), and 86 in the No-detected AF (no AF detected during the 2-year follow-up period) (Figure 1).

Consistent with the predefined primary objective, the main analysis focused on comparisons between the Late-detected AF and No-detected AF groups, while comparisons involving the Early-detected AF group were performed as exploratory analyses.

**Figure 2:**
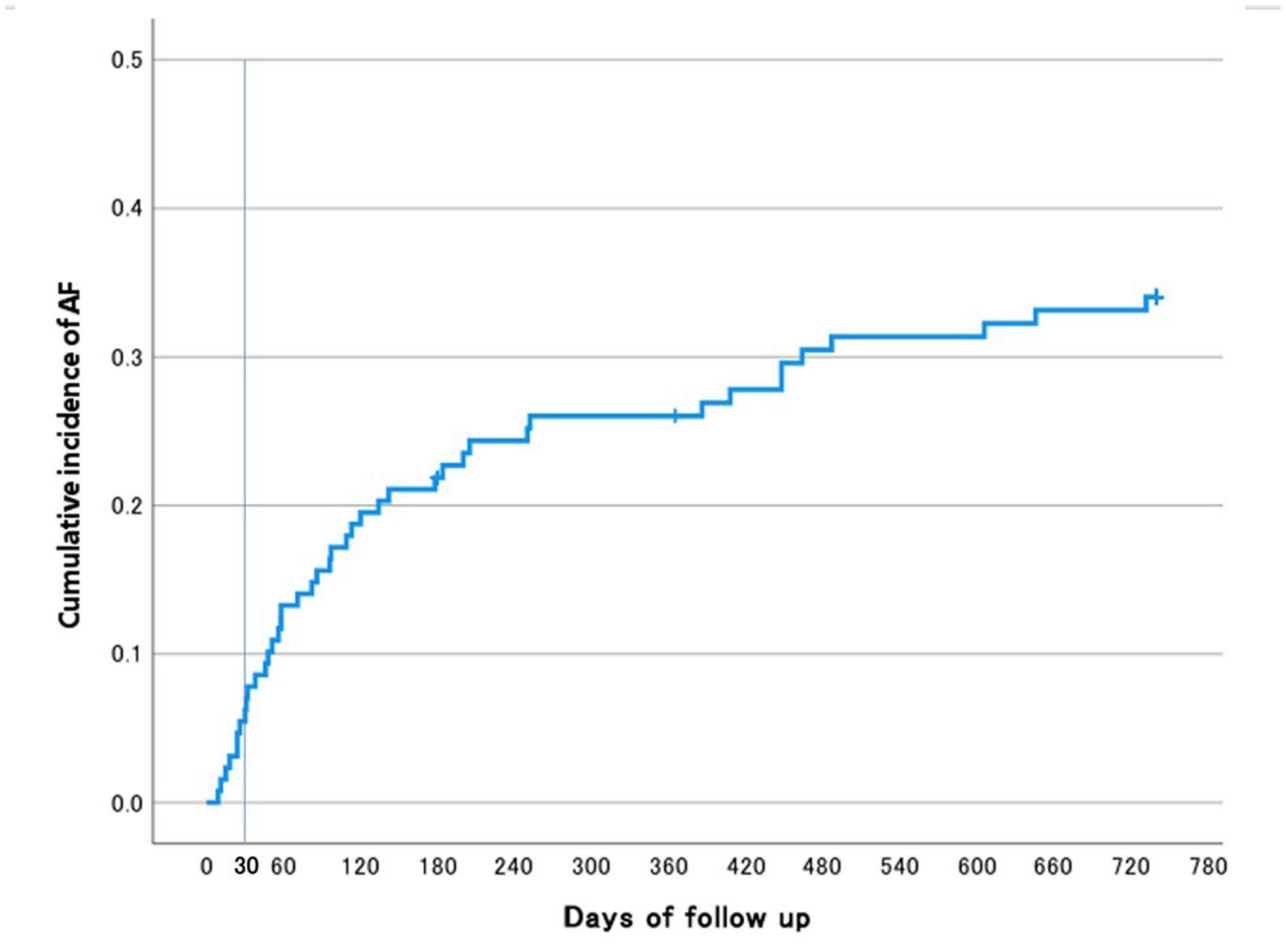
Kaplan–Meier curve for detection of atrial fibrillation. The Kaplan–Meier curve shows the cumulative incidence of atrial fibrillation (AF) detected by insertable cardiac monitors during the follow-up period. Among the 42 patients in whom AF was detected, 8 patients (19%) were diagnosed within 30 days of stroke onset, whereas 34 patients (81%) were diagnosed after 30 days. A larger proportion of AF cases were detected after 30 days; however, this observation should be interpreted with caution because of the longer observation period beyond 30 days.

As shown in Figure 2 (Kaplan–Meier curve), AF was detected in 8 of 42 patients (19%) within 30 days of stroke onset and in 34 of 42 patients (81%) after 30 days. A larger proportion of AF cases were detected after 30 days; however, this observation should be interpreted with caution given the longer observation period beyond 30 days.

Table 1 presents the characteristics of patients in the Late-detected and No-detected AF groups. Patients in the Late-detected AF group were older than those in the No-detected AF group (mean age (range): 78 [69–82] years vs. 66 [55–77] years, *p* < 0.001). Hyperlipidemia was less frequent in the Late-detected AF group (*p* = 0.03). Patients in the Late-detected AF group had LVO (9 [26.5%] vs. 9 [10.5%]; *p* = 0.003). D-dimer levels were significantly higher in the Late-detected AF group (1.1 [0.6–2.3] vs. 0.7 [0.6–1.3] μg/mL; *p* = 0.04). No significant differences were observed between the two groups for sex, other vascular risk factors, electrocardiographic findings, or echocardiographic parameters.

**Table 1:** Baseline characteristics of the study participants between late-detected AF and no-detected AF.

Variables	Late-detected AF	No-detected AF	*p*-value
	*n* = 34	*n* = 86	
Male sex, *n* (%)	22 (64.7)	61 (70.9)	0.52
Age, y	78 (69–82)	66 (55–77)	<0.01
Medical history, *n* (%)			
Hypertension	18 (52.9)	55 (64.0)	0.30
Hyperlipidemia	6 (17.6)	34 (39.5)	0.03
Diabetes mellitus	5 (14.7)	13 (15.1)	1.00
Congestive heart failure	1 (2.9)	2 (2.3)	1.00
Smoking	11 (32.4)	45 (52.3)	0.07
Prior ischemic stroke/TIA	5 (14.7)	19 (22.1)	0.45
Malignancy	2 (5.9)	6 (7.0)	1.00
CHADS_2_ score	4 (3–4)	3 (3–4)	0.91
CHA_2_DS_2_VASc score	5 (3–6)	4 (3–5)	0.38
Baseline NIHSS score	2 (1–7)	3 (1–5)	0.76
Score on modified Rankin scale	1 (1–2)	1 (0–2)	0.34
Days to insertion from onset	17 (13–18)	13 (11–16)	0.01
Radiological findings			
ASPECTS	10 (8–10)	10 (8–10)	0.63
Cortical lesion	25 (73.5)	69 (80.2)	0.47
Multiple territory lesions	14 (41.2)	30 (35.3)	0.68
Bilateral lesions	3 (8.8)	10 (11.6)	0.76
Large lesion	2 (5.9)	9 (10.5)	0.73
Prior cortical lesion	6 (17.6)	25 (29.1)	0.25
Large-vessel occlusion	9 (26.5)	9 (10.5)	0.03
Cardiothoracic ratio, %	53.9 (49.1–59.8)	51.9 (47.7–55.6)	0.11
Electrocardiographic findings			
P wave axis	42 (23–53)	41 (20–58)	0.93
PTFV_1_, μV*ms	2,480 (1120–5,016)	2040 (1190–4,000)	0.63
APC at 24 h	109 (16–291)	92 (29–504)	0.94
Total heartbeats at 24 h	89,185 (83478–99,350)	95,961 (85651–106,965)	0.11
Echocardiographic findings			
Left atrial appendaex flow velocity, m/s	0.5 (0.4–0.7)	0.5 (0.4–0.7)	0.66
Ejection fraction	65.5 (60.0–69.8)	65.0 (62.0–69.0)	0.68
Left atrial volume index, mL/m^2^	28.9 (23.3–33.0)	31.5 (24.2–41.4)	0.30
R-L shunt	3 (12.0)	19 (30.2)	0.10
Labo data			
Hemoglobin, g/dL	14.2 (13.4–15.3)	14.3 (13.5–15.4)	0.98
Platelet, ×10^4/μL	21.7 (16.9–26.6)	22.9 (17.9–25.8)	0.74
BNP, pg/mL	48.4 (16.9–105.8)	29.0 (14.2–54.8)	0.36
D-dimer, μg/mL	1.1 (0.6–2.3)	0.7 (0.6–1.3)	0.04
C-reactive protein, mg/dL	0.08 (0.04–0.33)	0.1 (0.05–0.23)	0.68

Values are mean ± SD, No. (%) or as otherwise indicated. TIA, transient ischemic attack; modified Rankin scale; NIHSS, National Institutes of Health Stroke Scale; ASPECTS, Alberta Stroke Program Early CT Score; PTFV_1_, P terminal force at V_1_; APC, Atrial Premature Contraction; BNP, Brain natriuretic peptide.

The results of the multiple logistic regression analyses of these factors are shown in Table 2. Variables with *p* < 0.05 in the univariate analysis were considered candidates for multivariable analysis. In this analysis, age (OR 1.067; 95% CI 1.025–1.117; *p* = 0.002), hyperlipidemia (OR 0.240; 95% CI 0.080–0.719; *p* = 0.011), and LVO (OR 4.137; 95% CI 1.301–13.153; *p* = 0.016) were significantly associated with late-detected AF.

**Table 2:** Multivariable predictors of late-detected AF.

Variables	OR (95%CI)	*p*-value
Age	1.067 (1.025–1.117)	0.002
Hyperlipidemia	0.240 (0.080–0.719)	0.011
Large-vessel occlusion	4.137 (1.301–13.153)	0.016

Table 3 shows a comparison of the clinical characteristics of the Late-detected AF (*n* = 34) and Early-detected AF (*n* = 8) groups. In the univariate analysis, no significant differences were observed between the two groups in terms of baseline demographics, vascular risk factors, or imaging findings. The modified Rankin Scale score at discharge tended to be lower in the Late-detected AF group, with borderline statistical significance (*p* = 0.05). Multivariate analysis was not performed owing to the small sample size (*n* = 8) of the Early-detected AF group.

**Table 3:** Baseline characteristics of the study participants between late-detected AF and early-detected AF.

Variables	Late-detected AF	Early-detected AF	*p*-value
	*n* = 34	*n* = 8	
Male sex, *n* (%)	22 (64.7)	4 (50.0)	0.45
Age, y	78 (69–82)	76 (75–85)	0.36
Medical history, *n* (%)			
Hypertension	18 (52.9)	5 (62.5)	0.63
Hyperlipidemia	6 (17.6)	1 (12.5)	1.00
Diabetes mellitus	5 (14.7)	1 (12.5)	1.00
Congestive heart failure	1 (2.9)	0 (0)	1.00
Smoking	11 (32.4)	3 (37.5)	1.00
Prior ischemic stroke/TIA	5 (14.7)	1 (12.5)	1.00
Malignancy	2 (5.9)	0 (0)	1.00
CHADS_2_ score	4 (3–4)	4 (3–4)	0.87
CHA_2_DS_2_VASc score,	5 (3–6)	5 (4–6)	0.49
Baseline NIHSS score	2 (1–7)	2 (1–2)	0.24
Score on modified Rankin scale	1 (1–2)	3 (1–3)	0.05
Days to insertion from onset	17 (13–18)	11 (10–16)	0.14
Radiological findings			
ASPECTS	10 (8–10)	10 (10–10)	0.14
Cortical lesion	25 (73.5)	6 (75.0)	0.93
Multiple territory lesions	14 (41.2)	5 (62.5)	0.28
Bilateral lesions	3 (8.8)	0 (0)	1.00
Large lesion	2 (5.9)	0 (0)	1.00
Prior cortical lesion	6 (17.6)	0 (0)	0.58
Large-vessel occlusion	9 (26.5)	0 (0.0)	0.17
Cardiothoracic ratio, %	53.9 (49.1–59.8)	56.5 (49.1–48.2)	0.87
Electrocardiographic findings			
P wave axis	42 (23–53)	37.5 (33.8–43.8)	0.97
PTFV_1_, μV*ms	2,480 (1120–5,016)	1,600 (1440–1800)	0.19
APC at 24 h	109 (16–291)	61 (32–156)	0.70
Total heartbeats at 24 h	89,185 (83478–99,350)	95,863 (87614–99,629)	0.50
Echocardiographic findings			
Left atrial appendaex flow velocity, m/s	0.5 (0.4–0.7)	0.5 (0.4–0.6)	0.77
Ejection fraction	65.5 (60.0–69.8)	66.5 (65.5–68.5)	0.40
Left atrial volume index, mL/m^2^	28.9 (23.3–33.0)	26.4 (24.8–27.8)	0.48
R-L shunt	3 (12.0)	0 (0.0)	1.00
Labo data			
Hemoglobin, g/dL	14.2 (13.4–15.3)	14.5 (14.0–14.7)	0.72
Platelet, ×10^4/μL	21.7 (16.9–26.6)	21.6 (18.5–25.5)	0.95
BNP, pg/mL	48.4 (16.9–105.8)	31.9 (14.8–38.5)	0.34
D-dimer, μg/mL	1.1 (0.6–2.3)	0.8 (0.6–0.8)	0.22
C-reactive protein, mg/dL	0.08 (0.04–0.33)	0.06 (0.03–0.22)	0.33

Values are mean ± SD, No. (%) or as otherwise indicated. TIA, transient ischemic attack; modified Rankin scale; NIHSS, National Institutes of Health Stroke Scale; ASPECTS, Alberta Stroke Program Early CT Score; PTFV_1_, P terminal force at V_1_; APC, Atrial Premature Contraction; BNP, Brain natriuretic peptide.

## 4 Discussion

AF detection was more frequently observed beyond 30 days after stroke onset than within the initial 30-day period; however, this finding should be interpreted cautiously because of the longer observation period after 30 days. Moreover, older age and the presence of LVO were associated with late AF detection compared with the no-detected AF group, whereas a history of dyslipidemia was negatively associated.

Because the primary objective of this study was to identify clinical characteristics associated with late-detected AF, the main analyses were focused on comparisons between the Late-detected and No-detected AF groups. In contrast, comparisons involving the Early-detected AF group were considered exploratory due to the limited sample size and were intended to generate hypotheses regarding timing-related differences. In comparisons between the late-detected AF and no-detected AF groups, older age and the presence of LVO were independently associated with late AF detection; however, a history of dyslipidemia showed a negative association. Older age is a well-established risk factor for AF and is included in several AF prediction scores for CS (14). Furthermore, age was identified as a key determinant of AF detection (11, 15–17). This finding may be explained by the age-related increase in AF prevalence (18). LVO in patients with CS is associated with a higher likelihood of subsequent AF detection (19). From previous studies, LVO has been strongly linked to cardioembolic stroke, including AF (20, 21). Accordingly, LVO in patients should prompt the consideration of AF detection, particularly with extended cardiac monitoring. Conversely, the absence of dyslipidemia may indicate a lower burden of atherosclerosis (22), which could, in part, be consistent with non-atherosclerotic embolic mechanisms.

Only minor differences were observed between the late- and early-detected AF groups, with the late AF group exhibiting lower preimplantation modified Rankin Scale scores. Furthermore, the clinical characteristics of the two groups were similar, suggesting fundamentally comparable clinical profiles. Relatively low modified Rankin Scale scores in the late AF group indicated mild initial symptoms. Previous studies have reported that CS and embolic stroke of undetermined source are associated with milder neurological deficits than cardiogenic cerebral embolism (23, 24), which is consistent with our findings. Such milder presentations may reduce clinicians’ suspicion of AF, resulting in less intensive early rhythm assessment. Therefore, even among patients with mild symptoms, active and sustained long-term follow-up is essential to avoid missing late-appearing AF and to optimize secondary stroke prevention, particularly in patients with high-risk features identified in this study, including advanced age and LVO. These findings have important implications for routine clinical practice, particularly for reducing the risk of recurrent cardiogenic cerebral embolism.

This study has certain limitations. First, the early-detected AF group was small, limiting the statistical power of the group comparisons. Second, causal inferences were not established in this observational study. Third, inter-individual variability in the timing of ICM implantation may have influenced the likelihood of AF detection. Additionally, some clinical variables were missing. Fourth, short AF episodes (<30 s) were not captured. Future studies should aim to develop prediction models for late-detected AF based on the identified factors and validate them in external cohorts.

## 5 Conclusion

AF detection after cryptogenic stroke frequently occurs following the early post-stroke period. Older age and LVO can identify patients at high risk of late-detected AF, underscoring the need for prolonged rhythm monitoring to optimize secondary prevention.

## Data Availability

The raw data supporting the conclusions of this article will be made available by the authors, without undue reservation.

## The LOOK investigators

The LOOK investigators’ affiliations are as follows: Department of Neurology, Nippon Medical School, Tokyo, Japan (Kutsuna, Suzuki, Sakamoto); Department of Neurosurgery, Yokohamashintoshi Neurosurgical Hospital, Kanagawa, Japan (Yamzaki); Department of Neurosurgery, Kawasakisaiwai Hospital, Kanagawa, Japan (Narikiyo); Department of Neurology, Saiseikai Fukuoka General Hospital, Fukuoka, Japan (Sonoda); Department of Neurology, the Jikei University School of Medicine, Tokyo, Japan (Komatsu); Division of Neurology, Department of Internal Medicine, Showa University School of Medicine, Tokyo, Japan (Mizuma); Department of Neurology, Showa University Koto Toyosu Hospital, Tokyo, Japan (Kuriki); Department of Neurology, Brain Attack Center Ota Memorial Hospital (Tesarawa); Department of Neurology, Japanese Red Cross Kumamoto Hospital (Terasaki); Division of Neurology, Department of Medicine, Jichi Medical University (Fujimoto); Department of Neurology, Juntendo University Faculty of Medicine (Miyamoto); Division of Neurology, Department of Internal Medicine, Toho University Faculty of Medicine (Kano, Nagasawa); Department of Neurology, Tokyo Women’s Medical University (Kitagawa), Department of Stroke Neurology, National Hospital Organization Osaka National Hospital/ Division of Stroke Prevention, Faculty of Medicine, University of Tsukuba (Yamgami); Department of Neurosurgery, Seisho Hospital, Kanagawa, Japan (Takeuchi); Department of Neurology, Dokkyo Medical University, Tochigi, Japan (Takekawa), Department of Cerebrovascular Medicine, NTT Medical Center Tokyo, Tokyo, Japan (Okubo); Department of Neurology, New Tokyo Hospital, Chiba, Japan (Shimoyama); Department of Neurology, Shioda Hospital, Chiba, Japan (Nomura); Department of Neurology, Ichinomyanishi Hospital, Aichi, Japan (Yamaguchi); Department of Neurology, Kobe City Medical Center General Hospital, Hyogo, Japan (Ohara).
